# Introduction of Mercury-Free Gold Extraction to Small-Scale Miners in the Cabo Delgado Province in Mozambique

**DOI:** 10.5696/2156-9614-8.19.180909

**Published:** 2018-09-10

**Authors:** Birgitte Stoffersen, Peter WU Appel, Leoncio D Na-Oy, Asta Selloane Sekamane, Ivan Zahinos Ruiz, Rasmus Køster-Rasmussen

**Affiliations:** 1 Diàlogos, Copenhagen, Denmark; 2 The Benguet Federation of Small-scale Miners, Itagon, Benguet, Philippines; 3 The Federation of Medicus Mundi, Barcelona, Spain; 4 The Research Unit for General Practice, University of Copenhagen, Denmark

**Keywords:** amalgamation, mercury, small-scale gold mining, borax, health, Mozambique

## Abstract

**Background.:**

The majority of small-scale gold miners worldwide, including those in Mozambique, use mercury to extract gold. Over the last fifty years, gold production from small-scale mining has been accelerating and consequently the amount of mercury released to the environment has increased dramatically, causing major global health problems. In 2018, a team from the Danish non-governmental organization Diálogos introduced the mercury-free gold extraction method in the Cabo Delgado province in Mozambique in the villages of Waqueia and Nanlia.

**Objectives.:**

The objective of this project was to teach local miners this method to reduce mercury pollution. An additional objective was to compare the local gold extraction method and the mercury-free gold extraction method in terms of gold recovery. The hypothesis was that the level of gold recovery would be higher with the mercury-free method compared to the locally used amalgamation method.

**Materials and Methods.:**

An experimental study comparing the two gold extraction methods was carried out where local miners processed gold-bearing ore using their standard procedures with the amalgamation method and the Diálogos team processed an equivalent amount of gold-bearing ore with the mercury-free gold extraction method. The tests were carried out once at each mining site.

**Results.:**

Under even circumstances in a controlled setting, the mercury-free method yielded up to 78% more gold than the amalgamation method normally used by the miners.

**Conclusions.:**

The strengths of the mercury-free gold extraction method include low costs, higher gold yield, benign environmental impact, legality and needed chemicals are more readily available compared with the amalgamation method. However, the mercury-free method may be more time consuming than the amalgamation method, especially for beginners. Borax is typically available in developed urban areas, as it is commonly used in the welding industry and by jewelers, but can be hard to find in more remote villages.

**Competing Interests.:**

The authors declare no competing financial interests.

## Introduction

Mercury is commonly used in small-scale gold mining to separate gold from other minerals due to its ability to bind to gold and form amalgam. Small-scale gold mining releases large amounts of mercury to the environment and is estimated to contribute approximately 37% of mercury emissions on a global scale.^1^ Mercury emissions have strong negative effects on the local environment, the health of the miners, as well as people living near the mines. In addition, mercury pollution affects the environment and human health on a global scale. In order to reduce global mercury pollution and the health hazards related to mercury emission, the use of mercury in small-scale mining should be stopped.^2^,^3^ International authorities such as the World Bank and the United Nations are working to address this issue and most countries have signed the Minamata convention aimed at reducing global mercury pollution.

Methods for gold extraction without the use of mercury are available. One method has proven to be implementable and safe.^4^ This method is called the ‘mercury-free gold extraction method’ and involves smelting with borax. Small-scale gold miners in Benguet in the northern Philippines invented the mercury-free gold extraction method. Large-scale mining companies later introduced the amalgamation method that uses mercury in the early 20th century. Small-scale miners felt that the amalgamation method was a more advanced method and began using it. However, it was not long before small-scale miners realized that the gold yield from the amalgamation method was lower than that of their traditional mercury-free method, which is the method currently used for gold extraction in Benguet. The mercury-free gold extraction method was implemented in the mining community Gaang in Northern Benguet in 2015.^4^ The community includes around 1800 small-scale miners that were formerly using the amalgamation method. The area remained mercury-free until at least 2017, when the last follow up was done, after the mercury-free gold extraction method was introduced.^4^

In February 2018, members of the Danish non-governmental organization (NGO) Diálogos and the Spanish NGO Medicus Mundi (funded by the European Union) carried out a project introducing the mercury-free gold extraction method by training a group of miners from the Cabo Delgado province in northern Mozambique. Teaching and training was carried out in the two mining communities of Waqueia and Nanlia. The objective of the present study was to compare the local gold extraction method and the mercury-free gold extraction method in terms of gold recovery and mercury consumption. The hypothesis was that the gold recovery would be higher with the mercury-free method compared to the locally used amalgamation method, as previous studies have suggested.^5^,^6^

## Materials and Methods

This was an experimental study comparing the gold yield from the amalgamation method and mercury-free gold extraction method. The study was carried out from February 11–19, 2018 at the mining sites of Waqueia and Nanlia in the Cabo Delgado province in northern Mozambique. For the comparison, local miners processed gold-bearing ore with their standard procedure using the amalgamation method and the Diálogos team processed an equivalent amount of gold-bearing ore with the mercury-free gold extraction method. To make an accurate comparison of the methods, the total amount of gold-bearing ore was divided in two portions, spade by spade, to ensure homogeneity in the two portions. The two portions were weighed to ensure that the amount of the lots was equal and the amount of gold in each lot was thought to be as identical as possible.

### Gold extraction methods

The default method for extraction of gold among small-scale mines in Mozambique is the amalgamation method.^7^ The process of amalgamation as carried out in the visited mines includes the following steps. First, the hard rock ore bodies are accessed from sinking shafts. The gold-bearing quartz veins are crushed into very coarse gravel and then ground into dry powder in a ball mill without adding water. The milled ore is subsequently washed down a chute covered with a piece of cloth. The cloth captures the grains of gold and other heavy minerals. The lighter minerals end up in the tailings (mine dumps). The concentrate of heavy minerals from the cloth is mixed with mercury in a washing pan, where the gold amalgamates with mercury. The amalgam is recovered and then heated over a bonfire in an iron cup. During heating, mercury evaporates and the gold remains, still containing some mercury. The gold yield from the amalgamation method is strongly dependent on processing techniques such as sluicing. The advantage of the amalgamation method is that it is easy and quick. The main disadvantage is the use of mercury and the fact that a good amount of gold is lost to the environment.^6^

**Figure 1:**
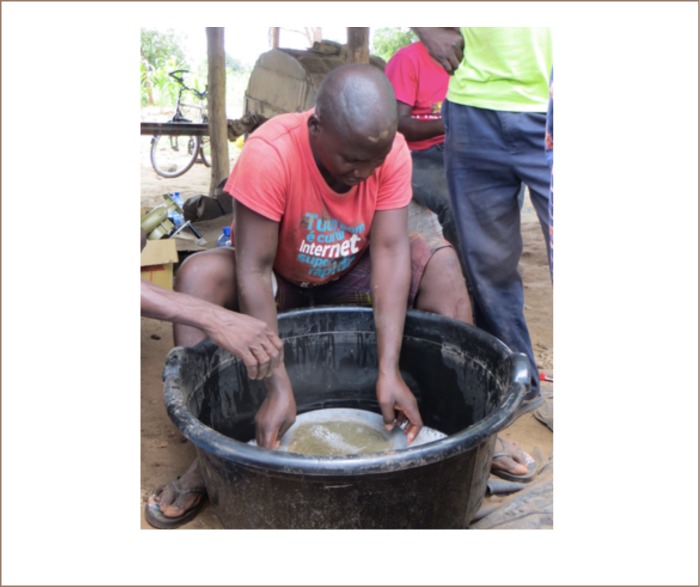
Local miner from Waqueia panning a heavy mineral concentrate

**Figure 2:**
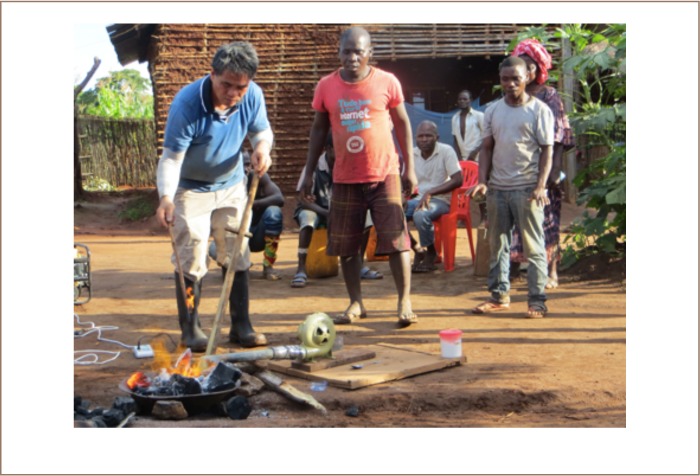
Gold smelting with borax

The mercury-free gold extraction method uses borax in the process of gold smelting. Borax is used in the smelting process because it lowers the melting point of gold and other metals. In this method, the mining of gold-bearing ore, the grinding of the mined material and the concentrating from sluicing are the same as in the amalgamation method. In order to achieve a high gold yield, it is important that the inclination of the chute does not exceed 20° and that the water flow is constant and not too fast for the cloth to be able to catch the heavy minerals. The concentrate of heavy minerals obtained from sluicing must contain a high concentration of gold for the borax to work as a flux to purify and smelt the gold concentrate.^6^ The concentrate is obtained through panning, where lighter minerals are discarded from the washing pan to the tailings due to gravity (*Figure 1*). The concentrate of heavy minerals contains magnetite, which is removed with a magnet. Gold is often trapped as fine grains within larger grains of heavy minerals.^7^ With the local amalgamation method, the fine gold is not liberated. To liberate the fine-grained gold, the heavy mineral concentrate is ground in the washing pan with a rock. The concentrate with a high proportion of gold is then mixed with borax in a small plastic bag. The amount of borax should not be less than 30% of the estimated gold dust weight. To smelt the gold, the mix is placed in a clay bowl with a few grams of borax and charcoal. The charcoal is fired up and high temperatures are obtained by using a manual or electrical blower. Depending on the intensity of the blowing the smelting of the gold takes 10–20 min, Figure 2.

### Comparison of the two methods

**Figure 3:**
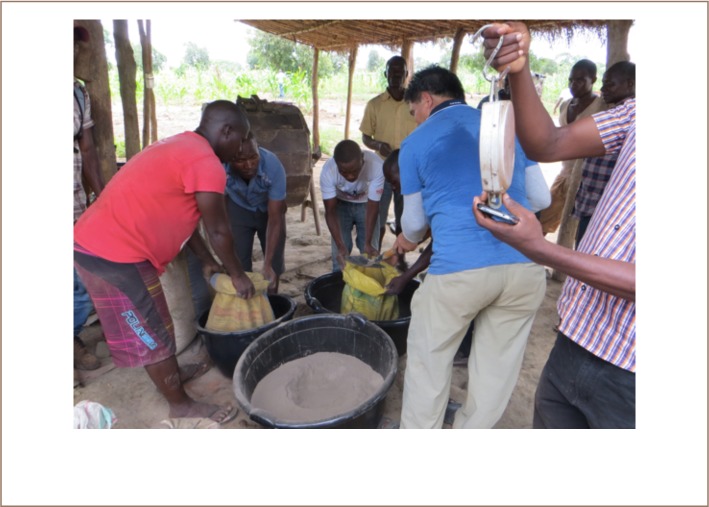
In order to compare the two methods, the milled ore was divided in two lots of equal amount and quality

**Figure 4:**
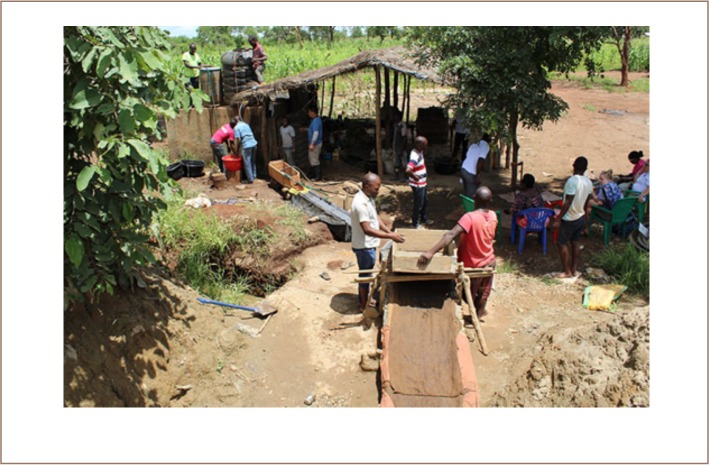
Waqueia. Foreground: processing station used for the amalgamation method Background: processing station used for the mercury-free gold extraction method

To compare the two methods and the quantitative outcome, two experiments were carried out; one in Nanlia and one in Waqueia. The first experiment was carried out in Waqueia on February 14, 2018, where 88 kg of milled ore was processed with each method. The ore was placed in a large tub, mixed and divided into two lots that were then weighed (*Figure 3*). The local processing set up was comprised of a chute made out of bamboo covered with old sacks and a sluice box (*foreground in Figure 4*) with holes approximately 1 cm in diameter. The local miners filled up the sluice box with the milled ore and washed it down the chute by adding water from buckets into the sluice box, which resulted in an uneven flow of water down the chute. Subsequently, the heavy mineral concentration caught by the sacks was washed into a tub, drained and placed in a washing pan, where mercury was added, forming an amalgam with the gold. The amalgam was separated from the concentrate and then put into an iron cup and heated over a bonfire. The processing set up used for the mercury-free gold extraction method also consisted of a chute and a sluice box (*background in Figure 4*). To process the ore, the milled ore was placed in the backend of the sluice box. The chute was covered with felt cloth. The milled ore was washed slowly down the chute with the use of a hose that was connected to a large barrel of water. The heavy mineral concentrate collected by the cloth was subsequently washed into a tub. The heavy mineral concentrate was then panned, the magnetite was collected with a magnet and the concentrate was ground with a rock to liberate the fine gold. The mineral concentrate was then mixed with borax and burned as described above. The second experiment was carried out in Nanlia on February 17, 2018. In Nanlia, 150 kg milled ore was processed with each method. The ore was divided spade by spade into two equal lots. The local processing set up in Nanlia was similar to the one in Waqeuia, except that the chute in Nanlia was covered with three layers: a layer of plastic on the bottom, followed by a layer of fabric and then a layer of cloth on top. The milled ore was placed in the sluice box and washed down the chute. The local miners in Nanlia used running water from a hose to create an even flow of water down the chute. A heavy mineral concentrate was collected from the layers of fabric and panned to further concentrate the ore. The mineral concentrate was then mixed with mercury to form an amalgam. The amalgam was heated in an iron cup. The Diálogos team used the same setup as in Waqueia as described above. The gold produced with the amalgamation method still contained some mercury after heating. In order to make a more accurate comparison, the gold produced with the amalgamation method was melted with borax to purify it and make the gold yield from the two methods comparable.

## Results

In Waqueia, the local miners recovered 0.9 g gold with the amalgamation method and the team from Diálogos recovered 1.6 g gold with the mercury-free gold extraction method. The mercury-free gold extraction method thus recovered 78% more gold than the amalgamation method. Extrapolating this result with information from the local miners regarding the yearly amount processed, the miners of Waqueia lose approximately 2 kg of gold every year plus substantial amounts of mercury.

In Nanlia, the two methods recovered equal amounts of gold (3.6 g).

## Conclusions

Under similar circumstances in a controlled but realistic setting, gold recovery with the mercury-free method was equal to or up to 78% higher than with the locally used amalgamation method.

A possible explanation for why the miners in Nanlia were able to get the same amount of gold from the milled ore as the Diálogos team was that the local miners used a very efficient sluicing technique and the ore contained no or very little fine gold and therefore not much gold was liberated from the gold concentrate when it was ground. Unlike the miners in Waqueia, the miners in Nanlia used a sluicing technique, where they were able to create an even flow of water, which allows fewer gold grains to escape the fabric on the chute. Furthermore, the miners in Nanlia used a finer woven cloth for their chute, which also allowed less gold to escape. The gold-bearing ore processed in Waqueia, on the other hand, contained fine grained gold that was liberated from the larger grains of heavy minerals.

The strength of the present study was that it was carried out in a real setting. The local miners used a well-known method in a familiar setting. This may have biased the results in favor of the amalgamation method. With further trials, the Diálogos miners may have further improved their gold yield with the mercury-free gold extraction method.

A limitation of the present study was that the comparison was only carried out two times. Further testing would have yielded more valid results. However, the experimental process is time consuming for researchers and gold miners, and the project was mainly an aid and technology transfer project, therefore it was not possible to allocate more time for further testing. Even after burning with borax, the gold produced with the amalgamation method can contain small amounts of mercury. Thus, the mercury-free method likely yielded a bit more gold even in Nanlia, where the methods at a first glance performed equally.

### Potential for implementation

Adopting the mercury-free gold extraction method requires time and practice, as the smelting procedure is more complex than burning amalgam. The miners were introduced to the economic and health advantages of the mercury-free gold extraction method as an incentive to change to the mercury-free method. The strengths of the mercury-free gold extraction method include low costs, higher gold yield, benign environmental impact, legality and needed chemicals are more readily available compared with the amalgamation method. However, the mercury-free method may be more time consuming than the amalgamation method, especially in the beginning. It was not possible to make a direct comparison of the time required for each method, as the test of the mercury-free gold extraction method was also a demonstration of the method, which made it more time consuming. Borax is typically accessible in developed urban areas, as it is commonly used in the welding industry and by jewelers, but can be hard to find in remote villages. As access to borax is essential for implementation of the mercury-free gold extraction method, establishment of borax distribution or information on distributors in remote villages would be beneficial.

### Recommendations

The goal of the present study was to persuade miners to adopt the mercury-free gold extraction method in the hopes that the new method would spread to other small-scale mining communities in the area. To this end, Diálogos members gathered with miners from Nanlia and Waqueia for discussions on how to disseminate the mercury-free gold extraction method to other groups in the Cabo Delgado province. The discussion focused primarily on barriers encountered by single miners or associations in their efforts to convince other miners or associations to go mercury-free. It was suggested that the associations could team-up and establish an umbrella federation. This could potentially represent thousands of miners and enhance access to the political system in order to improve working conditions for small-scale miners in Mozambique.
